# Cyst and endometriosis of the canal of Nuck: rare differentials for a female groin mass

**DOI:** 10.1093/jscr/rjab626

**Published:** 2022-01-22

**Authors:** Bridget Hwang, Jacqueline Bultitude, Jason Diab, Andrew Bean

## Abstract

Cyst of the canal of Nuck is a rare abnormality of the female inguinal canal that can present similarly to a hernia. If incompletely obliterated, the patent canal of Nuck may predispose to an inguinal hernia or hydrocele due to direct communication with the abdominal cavity. Such defects are normally detected and repaired in early childhood but can also present later in adult life. We report the case of a 44-year-old woman who presents with a fluctuant, mobile and irreducible left-sided groin mass. Ultrasound of the groin identified a cystic structure in the canal of Nuck. The patient underwent successful open herniorrhaphy with excision of the cyst and mesh repair of the inguinal canal. Subsequent histopathological examination also revealed concurrent endometriosis of the canal of Nuck. A systematic approach to differential diagnoses for a female groin mass, further investigations and management are discussed.

## INTRODUCTION

A groin mass, referring to swellings located in the inguinofemoral region at the junction of the upper leg and lower abdomen, is a very common presentation. The clinical features of a groin mass vary in size, shape, tenderness and consistency, reflecting various possible underlying pathological processes, including hernias, infection, neoplasms, vascular or congenital abnormalities [1]. While a comprehensive history and examination are often sufficient in making the diagnosis of an inguinal mass, ultrasonography is a useful first-line imaging modality to visualize local anatomy in order to keep a broader perspective of the differentials. We report the case of a 44-year-old woman who presents with a groin mass that is identified to be a cyst of the canal of Nuck (CCN). We present a systematic approach to differential diagnoses for a groin mass in a female patient and the distinguishing features of a CCN compared to other masses of the inguinofemoral region.

## CASE REPORT

A 44-year-old female presented to her general practitioner with a 1-month history of a left-sided groin mass. The mass was occasionally painful and was more prominent when standing and straining. It was not associated with any abdominal or pelvic pain, vomiting, constipation, dysuria or vaginal discharge. She was otherwise in good health with a background history of stage 3 cervical intraepithelial neoplasia, which was treated with large loop excision of the transformation zone. Her surgical history included two cesarean sections. On examination, the mass was lateral to the left pubic tubercle and measured ~2 cm in diameter. It was mobile, fluctuant and irreducible. Although it was more prominent upon standing, there was no cough impulse. The overlying skin was not erythematous and there was no associated local lymphadenopathy. The abdominal and vaginal examinations were unremarkable.

**Figure 1:**
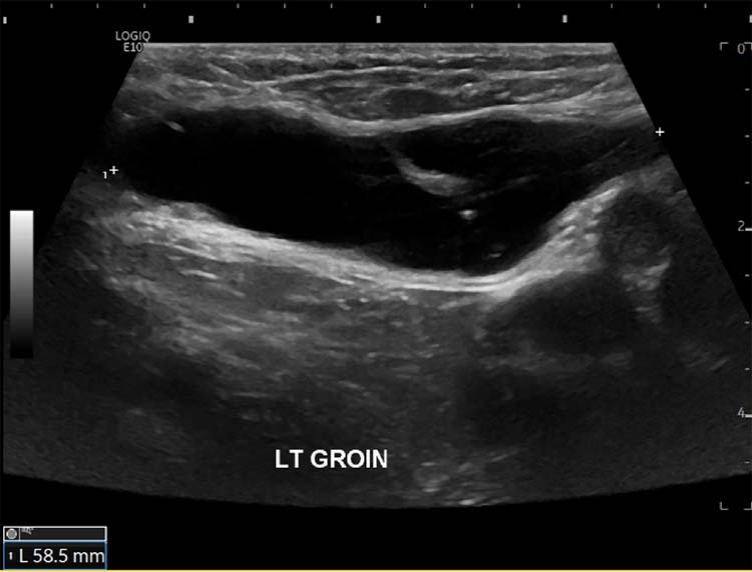
US scan of left groin showing a 59 × 25 × 13 mm anechoic thin-walled cystic structure in the canal of Nuck.

**Figure 2:**
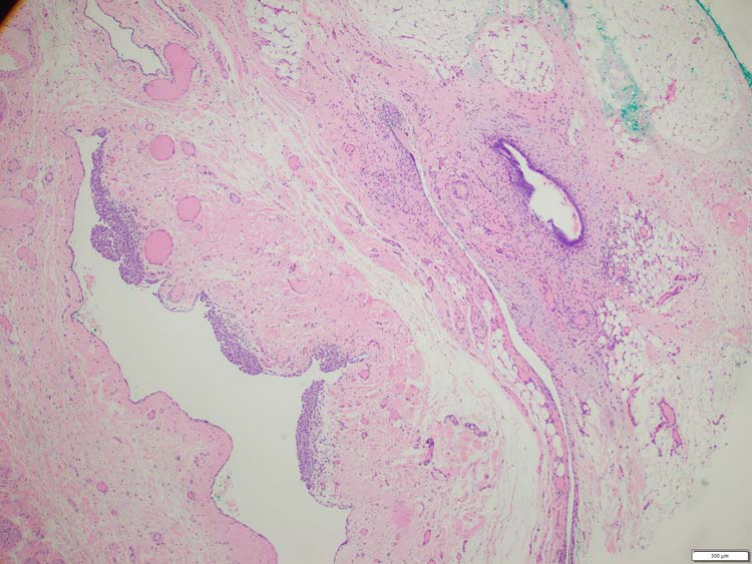
Histology of the surgical specimen (×40 magnification) shows a cystic structure lined in part by cuboidal epithelium in keeping with a cyst of canal of Nuck; focal endometriosis is identified in the adjacent soft tissue.

The patient was referred for an ultrasound (US) of the left groin, which showed a contained cyst in the inguinal canal consistent with a CCN (Fig. 1). She was referred to a general surgeon for elective surgical management. A herniorrhaphy was performed with excision of the canal of Nuck and mesh repair. The procedure occurred without complication and the patient was discharged the following day. Histopathology report confirmed a CCN as well as evidence of endometriosis in the adjacent tissue (Fig. 2).

## DISCUSSION

Groin masses in females are less widely encountered than in males due to key anatomic variations of the inguinal canal between sexes. Notably, males have a larger and more prominent inguinal canal, containing the spermatic cord and obliterated processus vaginalis, which accompanied the descent of the testes into the scrotum. This descent may leave an opening in the abdominal wall through which males are more susceptible to an indirect inguinal hernia [2]. On the other hand, the female inguinal canal is smaller and instead contains the round ligament of the uterus and the canal of Nuck, a small tubular evagination of parietal peritoneum that traverses through the female inguinal canal anterior to the round ligament and inserts into fibers of the labia majora. It can be considered the female equivalent of the processus vaginalis in males [3, 4]. Incomplete obliteration of the canal of Nuck during development may predispose to an indirect inguinal hernia or hydrocele due to direct communication with the abdominal cavity [3]. Such defects are generally detected and repaired in early childhood [4]. The exact incidence of a CCN or female hydrocele in the adult population, however, is unknown due to frequent misdiagnosis [5]. Other differential diagnoses for a female inguinofemoral mass can be categorized into hernias, infectious, neoplastic, vascular or congenital pathologies (Table 1) [1].

**Table 1:** Typical features of differential diagnoses for a female inguinofemoral mass

Category	Differential diagnosis	Typical features
Hernia	Femoral hernia	• A mass below the inguinal ligament, lateral and inferior to pubic tubercle
	Indirect inguinal hernia	• A mass originating above the mid-inguinal point • No protrusion of a reduced mass upon occlusion of the deep ring
	Abdominal wall hernias	• Spigelian, incisional or divarication of rectus
	Obturator hernia	• Elderly, emaciated females are a high-risk group • May present without a mass but with symptoms of bowel obstruction
Cyst	CCN (female hydrocele)	• A groin swelling that protrudes upon standing and disappears while lying • Negative cough impulse
	Cystic adenomyosis	• Severe inguinal menstrual pain • Nodule enlargement
	Bartholin’s cyst	• Found lower in the labial region
Abscess	Psoas abscess or abscess secondary to incarcerated hernia, perforated hernia, diverticulitis, etc.	• Painful with overlying skin erythema • Fever • Associated with groin lymphadenopathy
Vascular	Round ligament varicosities	• Often occurs during pregnancy • Negative cough impulse
	Femoral artery aneurysm	• A pulsatile mass may be iatrogenic following vascular procedure • Often occurs in the elderly
	Saphenous varix	• Dilatation of the saphenous vein at the saphenofemoral junction • Presents concurrently with varicosities of the lower limb(s)
	Hematoma	• History of trauma, surgery, neoplasm, catheterization or anticoagulation
Neoplasms	Lipoma	• Often asymptomatic • A soft, subcutaneous mass that does not change in size
	Liposarcoma	• A slow-growing, indolent soft tissue mass that is often painless
	Inguinal canal endometriosis	• Associated with symptoms of endometriosis, e.g. dysmenorrhea, dyspareunia
	Lymphoma	• A firm, fixed mass that can increase in size • Associated constitutional symptoms
Lymphadenopathy	Infectious	• Enlarged, tender, firm and mobile nodes • Usually multiple lymph nodes
	Malignant	• Constitutional symptoms • Presence of lower limb, genital or perianal primary malignancy

Clinically, CCN presents as a palpable, fluctuant, reducible or irreducible swelling in the inguinolabial region, which can be either painless or moderately painful [6]. Omental and intestinal contents may also herniate through a patent canal of Nuck, and approximately one-third of patients with a CCN can have a concurrent inguinal hernia requiring simultaneous repair [5]. Additionally, a patent canal of Nuck is a rare but potential location for seeding of endometrial tissue, with a prevalence of 0.3–0.6% of all endometriosis cases [7]. Theories for extra-pelvic endometriosis include retrograde menstruation, hematogenous or lymphatic spread or coelomic metaplasia [7, 8]. A female groin mass implicated with endometriosis is more likely to present with cyclical pain consistent with menstruation.

The inconsistent clinical presentation of the more unusual groin mass differentials poses a diagnostic challenge; therefore, ultrasonography is recommended as the first-line imaging modality as it can accurately differentiate between cystic or solid lesions. US appearance typically shows an oval thin-walled cystic lesion with smooth borders and without internal vascularity [5, 9] (Fig. 1). CT and magnetic resonance imaging are more useful alternatives if there are concerns of malignancy; however, US remains a more time- and cost-effective option that is not associated with radiation toward pelvic organs. Ultimately, intra-operative excision of the cyst and histopathology is required to confirm the diagnosis [10].

Surgery is often recommended even for an asymptomatic mass due to the strong association between CCN and inguinal hernias [9]. Referral to a general surgeon for elective open herniorrhaphy with excision of the cyst and mesh repair of the inguinal canal is recommended for definitive management. Laparoscopic techniques may be preferred by some surgeons; however, this could result in a longer and more difficult operation if the inguinal canal is deep or the CCN is too large, requiring conversion to an open approach [11]. Aspiration of the cyst is rarely curative and should be avoided as rate of recurrence is high. Recurrence following surgical repair, if at all, is a late complication, with most case reports describing no recurrence of a CCN within 2–24 months [5].

## Conclusion

In conclusion, a female inguinofemoral mass is a common presentation with a wide variety of differential diagnoses. CCN should be considered due to its similar presentation and close correlation with inguinal hernias. Endometriosis of the canal of Nuck or inguinal canal is also possible and is confirmed upon histology. Ultrasonography is the first-line imaging modality in distinguishing between solid and cystic groin masses before referring for surgical management.
